# Meta-Analysis Combines Affymetrix Microarray Results Across Laboratories

**DOI:** 10.1002/cfg.460

**Published:** 2005-04

**Authors:** John R. Stevens, R. W. Doerge

**Affiliations:** 1 Department of Statistics Purdue University 150 N. University Street West Lafayette IN 47907-2067 USA; 2 Department of Agronomy Lilly Hall of Life Sciences 915 W. State Street Purdue University West Lafayette IN 47907-2054 USA

## Abstract

With microarray technology becoming more prevalent in recent years, it is now common for several laboratories to employ the same microarray technology to identify differentially expressed genes that are related to the same phenomenon in the same species. Although experimental specifics may be similar, each laboratory will typically produce a slightly different list of statistically significant genes, which calls into question the validity of each gene list (i.e. which list is best). A statistically-based meta-analytic approach to microarray analysis systematically combines results from the different laboratories to provide a single estimate of the degree of differential expression for each gene. This approach provides a more precise view of genes that are of significant interest, while simultaneously allowing for differences between laboratories. The widely-used Affymetrix oligonucleotide array and its software are of particular interest because the results are naturally suited to a meta-analysis. A simulation model based on the Affymetrix platform is developed to examine the adaptive nature of the meta-analytic approach and to illustrate the utility of such an approach in combining microarray results across laboratories.


**Comparative and Functional Genomics**
*Comp Funct Genom* 2005; **6**: 116–122.
Published online in Wiley InterScience (www.interscience.wiley.com). **DOI:** 10.1002/cfg.460

**Conference Paper**

### Meta-analysis combines Affymetrix microarray results across laboratories

John R. Stevens[1] and R. W. Doerge[1,2]*
[1]*Department of Statistics, Purdue University, 150 N. University Street, West Lafayette, IN 47907-2067, USA*
[2]*Department of Agronomy, Lilly Hall of Life Sciences, 915 W. State Street, Purdue University, West Lafayette, IN 47907-2054, USA*

**Correspondence to:*
*R. W. Doerge, Department of Statistics, Purdue University, 150 N. University Street, West Lafayette, IN 47907-2067, USA.*
*E-mail: doerge@purdue.edu*

Received: 13 January 2005
Accepted: 19 January 2005

### Abstract

**With microarray technology becoming more prevalent in recent years, it is now common for several laboratories to employ the same microarray technology to identify differentially expressed genes that are related to the same phenomenon in the same species. Although experimental specifics may be similar, each laboratory will typically produce a slightly different list of statistically significant genes, which calls into question the validity of each gene list (i.e. which list is best). A statistically-based meta-analytic approach to microarray analysis systematically combines results from the different laboratories to provide a single estimate of the degree of differential expression for each gene. This approach provides a more precise view of genes that are of significant interest, while simultaneously allowing for differences between laboratories. The widely-used Affymetrix oligonucleotide array and its software are of particular interest because the results are naturally suited to a meta-analysis. A simulation model based on the Affymetrix platform is developed to examine the adaptive nature of the meta-analytic approach and to illustrate the utility of such an approach in combining microarray results across laboratories.** **Copyright © 2005 John Wiley & Sons, Ltd.**

**Keywords:** **meta-analysis; microarray; differential expression**

### Introduction

When a laboratory employs microarray technology to identify genes related to a specific disease or condition of interest, the published result is most often a list of candidate genes determined to be statistically significant. This claim of significance is usually based on some consideration of a measure of differential expression between a control (non-diseased) sample and a treatment (diseased) sample, and should be based on a statistical test to account for variability in the experimental data. Although there are numerous measures of differential expression and statistical tests found in an ever-growing body of literature [7–9,16], the results are often vastly different. Even when multiple laboratories investigating the genetic basis of the same biological phenomenon in the same organism, using essentially the same experimental design and microarray platform, report differential expression using the same measure and statistical test, the lists of candidate genes from each laboratory may differ considerably.

In an attempt to draw together the results from each laboratory while acknowledging their differences, we applied a meta-analytic approach [24], which considers each laboratory as a separate study investigating the same effect. The results from each laboratory are combined in a systematic manner to arrive at a more precise understanding of each gene's true relationship to the phenomenon of interest. Where the term 'analysis' is used to describe the quantitative approaches to draw useful information from raw data, the term 'meta-analysis' [13] refers to the approaches used to draw useful information from the results of previous analyses. Such approaches have particular utility with the results from Affymetrix GeneChip® microarrays and other

Copyright © 2005 John Wiley & Sons, Ltd.

fabricated arrays, because these results are given in a uniform format and readily lend themselves to comparisons between labs. Of greatest interest are the meta-analytic approaches that yield not only a list of candidate genes based on statistical tests, but also an estimate (and associated standard error) of the degree or magnitude of differential expression. This information is useful not only in identifying which genes are differentially expressed, but also in making statements about the degree to which they are differentially expressed between the tested conditions.

Recently, several applications of meta-analysis to microarray data have appeared in the literature [5,12,17–21,26]. Most of these approaches have focused on combining significant results rather than on providing estimates of the magnitude of differential expression. Those that have provided quantitative estimates focused on combining results across platforms without fully accounting for the technological differences. An approach that *combines the results* from microarray experiments to produce a single estimate of the degree or magnitude of differential expression for each gene is presented here.

### Differential expression with Affymetrix microarray technology

The Affymetrix GeneChip® microarray [1] is currently one of the most widely-used platforms for gene expression experiments. Affymetrix has developed statistical algorithms [23] that employ individual spot intensities on the microarray for the purpose of estimating the true expression levels of individual genes in single samples. Furthermore, the Affymetrix software MAS 5.0 [2] compares gene expression levels in two different tissues (samples or treatment conditions) and reports a 'signal log ratio' (SLR) with 95% confidence bounds. The signal log ratio is the signed $\log_2$ of the signed fold change (FC) familiar to biologists [25], i.e. $\mathrm{FC} = 2^{\mathrm{SLR}}$ if $\mathrm{SLR} > 0$ and $\mathrm{FC} = (-1)2^{-\mathrm{SLR}}$ if $\mathrm{SLR} < 0$.

If we let $\tilde{\theta}_{i,k}$ denote the estimate of the SLR $\theta_{i,k}$ for gene $k$ in lab $i$, and $\tilde{\theta}_{i,k}^{upper}$ be the upper bound for the 95% confidence interval, then both $\tilde{\theta}_{i,k}$ and $\tilde{\theta}_{i,k}^{upper}$ are reported by the Affymetrix software. The Affymetrix documentation [23] gives the estimated standard error of $\tilde{\theta}_{i,k}$ as $s_{i,k} = (\tilde{\theta}_{i,k}^{upper} - \tilde{\theta}_{i,k})/t_{i,k}^{(0.975)}$, where $t_{i,k}^{(0.975)}$ is the upper 0.025 critical value of the $t$ distribution with $df_{i,k} = \max(0.7(n_{i,k} - 1), 1)$ degrees of freedom, where $n_{i,k}$ represents the number of probe pairs representing gene $k$ on each array in study $i$. Based on this, lab $i$ may declare gene $k$ significantly differentially expressed if zero is not within the confidence interval $\tilde{\theta}_{i,k} \pm s_{i,k} t_{i,k}^{1-\alpha/2}$, with $\alpha$ appropriately selected to adjust for multiple comparisons. The estimated variance of the SLR estimate $\tilde{\theta}_{i,k}$ is $v_{i,k} = s_{i,k}^2$.

In order to be combined across studies, quantitative estimates must address the same measure or quantity, be standardized to the same scale, and include some measure of variability [14]. The SLR satisfies these criteria. Specifically, the SLR for a given gene represents the degree of differential expression between two conditions and is directly comparable between labs, since it estimates the same physical quantity. The SLR from Affymetrix is standardized in the sense that a SLR of zero means no differential expression is observed, and the algorithms used to produce the SLR place all SLR estimates on the same scale. A variance for the SLR estimate is provided by the Affymetrix algorithms. In fact, the Affymetrix software MAS 5.0 [2,23] gives the results from each laboratory in a uniform format, providing the necessary components for a meta-analysis: a quantitative estimate ($\tilde{\theta}_{i,k}$) and the associated measure of variability ($v_{i,k}$).

### Random effects meta-analysis

Three main meta-analytic approaches exist [6]: fixed effects, random effects and hierarchical Bayes. The first two will be summarized here, focusing on the random effects model [10]; the third is beyond the scope of this review. The basic meta-analytic model can be represented as:

$$\tilde{\theta}_{i,k} = \theta_{i,k} + \varepsilon_{i,k} = \theta_k + \delta_{i,k} + \varepsilon_{i,k}$$

where $\tilde{\theta}_{i,k}$ is the observed SLR estimate for gene $k$ from experiment $i$ ($i = 1, \ldots, n$), and $\varepsilon_{i,k}$ is the within-experiment sampling error. Here, $\theta_k$ is the quantity that experiment $i$ is expected to measure (the true underlying SLR for gene $k$), but due to the random deviation $\delta_{i,k}$, experiment $i$ is in fact measuring $\theta_{i,k}$. That is, there is a true SLR

Copyright © 2005 John Wiley & Sons, Ltd. 

($\theta_k$) for gene $k$ but, due to random deviation, the SLR in each experiment ($\theta_{i,k}$ in experiment $i$) is slightly different. It is typically assumed that $\delta_{i,k}$ is normally distributed with mean zero and variance $\Delta_k^2$, and $\varepsilon_{i,k}$ is normally distributed with mean zero and variance $\sigma_{i,k}^2$. The variance $\sigma_{i,k}^2$ is estimated by $v_{i,k}$, the estimated variance of the observed SLR.

The fixed effects meta-analysis model carries the additional assumption that $\Delta_k^2 = 0$. This assumption, referred to as the homogeneity assumption, has the interpretation of assuming that gene $k$ is expressed the same in all experiments, and that observed differences are due to sampling error only. In practice, the fixed effects model tends to be overly simplistic and will not be considered further in this review.

The quantity of interest in this model is $\theta_k$, the underlying 'true' SLR value for gene $k$. The method of moments [6] can be used to estimate $\Delta_k^2$ and then $\theta_k$ by following a generalized least squares approach [22]. First, let $Q_k = \sum w_{i,k}(\tilde{\theta}_{i,k} - \theta_k^*)^2$, where $w_{i,k} = 1/v_{i,k}$ and $\theta_k^* = \sum w_{i,k}\tilde{\theta}_{i,k} / \sum w_{i,k}$. Note that $\theta_k^*$ is a weighted average of the observed SLR estimates $\tilde{\theta}_{i,k}$, with weights $w_{i,k}$ selected to minimize the variance of $\theta_k^*$. Then estimate $\Delta_k^2$ by [10]:

$$\hat{\Delta}_k^2 = \max\left(0, \frac{\sum w_{i,k}(Q_k - n + 1)}{\left(\sum w_{i,k}\right)^2 - \sum w_{i,k}^2}\right)$$

and compute adjusted weights $\tilde{w}_{i,k} = (v_{i,k} + \hat{\Delta}_k^2)^{-1}$.

The estimate of $\theta_k$ is $\hat{\theta}_k = \sum \tilde{w}_{i,k}\tilde{\theta}_{i,k} / \sum \tilde{w}_{i,k}$ with estimated variance $\hat{v}_k = \left(\sum \tilde{w}_{i,k}\right)^{-1}$. To test the significance (i.e. the significant differential expression) of gene $k$, the test statistic $Z_k = \hat{\theta}_k / \sqrt{\hat{v}_k}$ is employed. Under $H_{0,k} : \theta_k = 0$, $Z_k$ approximately follows the standard normal distribution, therefore gene $k$ can be assigned a $P$ value, $P_k = 2(1 - \Phi(|Z_k|))$, where $\Phi$ is the cumulative distribution function of the standard normal distribution. The meta-analysis declares gene $k$ significantly differentially expressed at significance level $\alpha$ if $P_k < \alpha$, where $\alpha$ may be appropriately adjusted for multiple comparisons.

### Simulation model

In order to evaluate the utility and power of the proposed meta-analytic approach, a simple simulation study was conducted. It is useful to use the results from a simulation study to compare with the true simulation setting, or the 'truth', for the purpose of assessing how well the method works (i.e. in this case, the meta-analysis). Specifically, the adaptive nature of the meta-analytic approach can be illustrated by comparing the 'true' SLR values with the estimates from each simulated lab and from the meta-analysis.

'Raw' probe-level data were generated from a model assuming that mismatch intensities (MM) are random background noise, which is an underlying assumption of the Affymetrix approach [23]. Specifically, the model assumes that the mismatch ($MM$) intensity for probe $l$ of gene $k$ under treatment $j$ in experiment $i$ followed a gamma distribution: $MM_{ijkl} \sim \Gamma(\alpha, \beta)$. Once the background mismatch intensities were obtained, the perfect match ($PM$) intensities were generated via the model:

$$\begin{aligned}\log_2(PM_{ijkl} - MM_{ijkl}) = \mu &+ L_i + G_k \\ &+ P(G)_{(k)l} + LG_{ik} \\ &+ \rho_k(T_j + LT_{ij} + TG_{jk} \\ &+ LTG_{ijk} + TP(G)_{j(k)l}) \\ &+ \varepsilon_{(ijk)l}.\end{aligned}$$

Six experiments were considered with each using the same two treatments (control and experimental). The term $\rho_k \sim \text{Bernoulli}(p)$ is 1 if gene $k$ is differentially expressed between conditions $j = 1$ and $j = 2$, and is 0 otherwise. The parameter $p$ corresponds to the percentage of genes that are differentially expressed, with higher values resulting in more differentially expressed genes. In this model, $L_i$ is the effect of lab $i$, $T_j$ is the effect of treatment $j$, $G_k$ is the effect of gene $k$, $P(G)_{(k)l}$ is the effect of probe $l$ of gene $k$, $\varepsilon_{(ijk)l}$ is a random error term, and the other terms are interaction effects. To introduce more between-lab variability, the error variance was allowed to be different in each lab. Various sources of variability in the 'observed' simulated data can be introduced by adjusting the parameters in this model.

Copyright © 2005 John Wiley & Sons, Ltd. 

### Results

Both the simulation and the meta-analysis were conducted using R software [15,25], as well as the affy package [11] from the BioConductor project [4,25]. The simulation was performed based on the Affymetrix rat neuro-chip RN_U34, with model parameter settings selected to produce a distribution of *MM* intensities similar to that observed in real data (Figure 1a, b) and to produce signal log ratio (SLR) estimates within a reasonable range and with some variation between laboratories (Figure 1c, d).

A random effects meta-analysis combined the SLR estimates from the six simulated labs to arrive at a single SLR estimate for each gene. The test of significance $H_{0,k} : \theta_k = 0$ was performed for all 1322 genes, and the $P$-values for each of the genes were summarized in a histogram of significance $P$-values (Figure 2a). If there were no differentially expressed genes (i.e., if $H_{0,k} : \theta_k = 0$ were true for all genes $k$), then this histogram should be relatively flat. The abundance of smaller $P$-values is indicative of a larger number of significant genes. These smaller $P$-values tended to correspond to the largest meta-analysis SLR

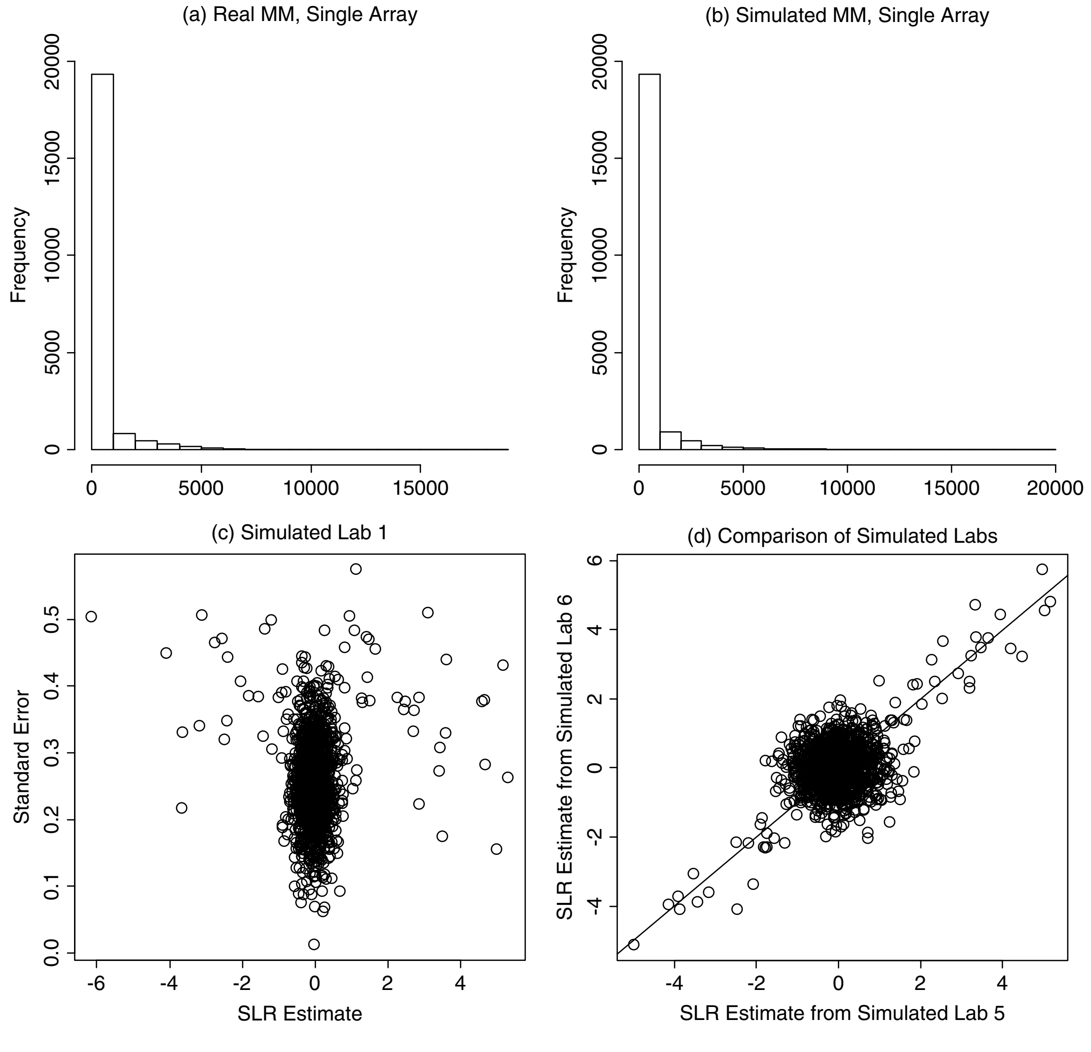

**Figure 1.** Summary of results from simulated labs. The simulation parameters were selected to force the simulated mismatch (*MM*) intensities (b) to have a distribution similar to that observed in real data (a). The SLR estimates from each lab (c) tended to be close to zero, with deviations from zero indicating differential expression. The simulated results from different labs (d) tended to be similar but also exhibited noticeable differences

Copyright © 2005 John Wiley & Sons, Ltd. 

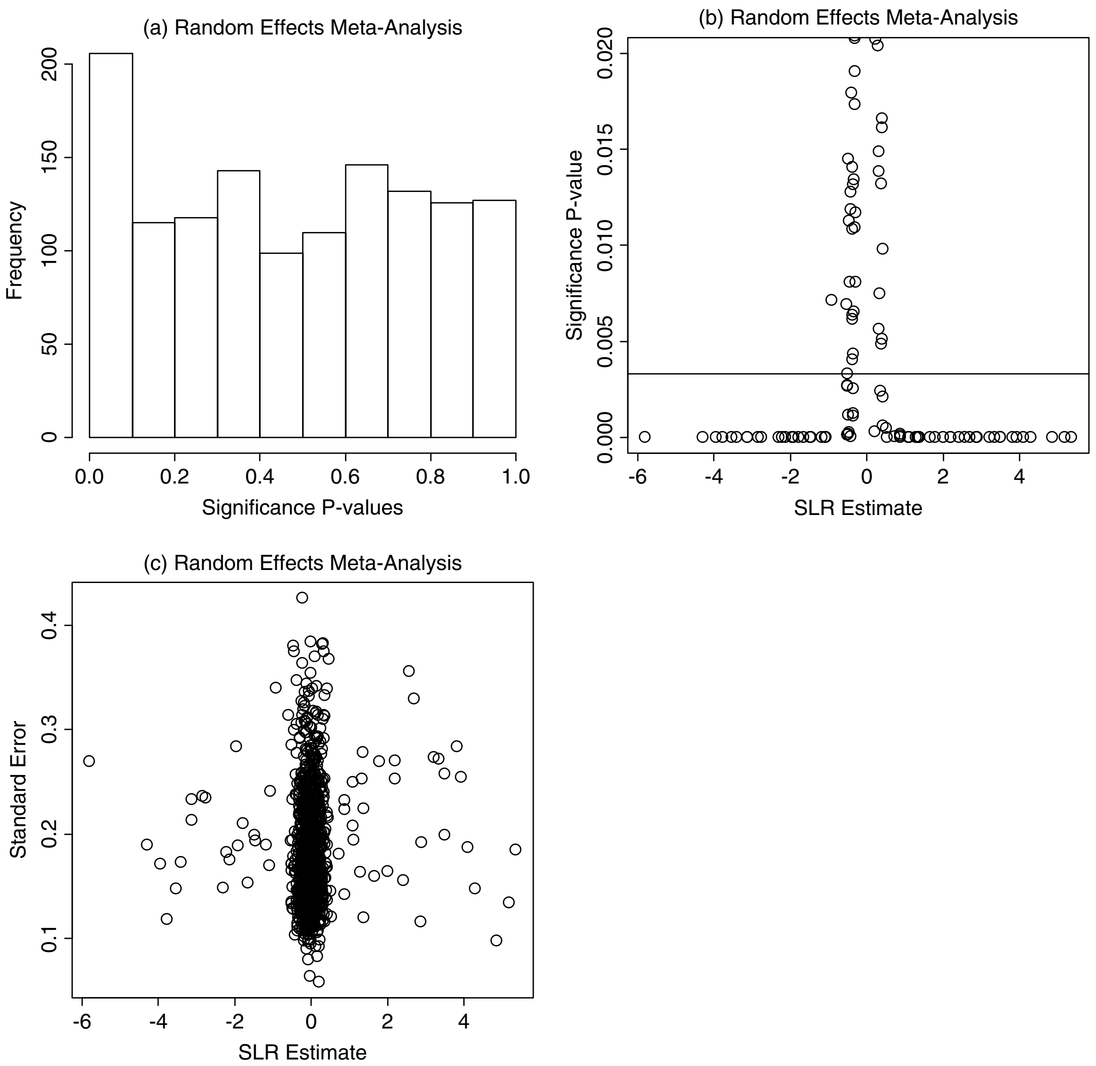

**Figure 2.** Summary of random effects meta-analysis. If there were no differentially expressed genes, the histogram of significance *P* values (a) would be expected to be relatively flat. The excess of small *P* values here indicates a large number of differentially expressed genes. Those genes with larger SLR estimates tended to have smaller significance *P* values (b) because larger SLR values are indicative of differential expression. The horizontal reference line in (b) represents the *P* value threshold to control the FDR at 0.05. The standard errors of the random effects meta-analysis SLR estimates (c) tend to be lower overall than the standard errors from any single lab (Figure 1c), particularly for genes with SLR values away from zero estimates (Figure 2b). Similar to the results from a single lab (Figure 1c), most meta-analysis SLR estimates were close to zero (Figure 2c; indicative of non-differential expression), but the standard errors were slightly lower overall for the meta-analysis estimates, after combining the SLR estimates across labs.

Under a random effects meta-analysis with the false discovery rate (FDR) [3] controlled at 0.05, 72 of the 1322 genes were declared significantly differentially expressed (i.e. $H_{0,k}$ : $\theta_k = 0$ is rejected). Individually, the six labs identified between 44 and 58 significantly differentially expressed genes (controlling the FDR at 0.05 for each lab) (Table 1). For each lab, most (but not all) of its significant genes were declared significant by the random effects meta-analysis.

The results from this small simulation demonstrate how a meta-analysis handles discrepancies between labs. The meta-analytic approach proved

Copyright © 2005 John Wiley & Sons, Ltd. 

**Table 1.** A summary of the number of common genes that are declared significantly differentially expressed by simulated labs 1–6 and the random effects meta-analysis

| | Simulated lab | | | | | | | |
|---|---|---|---|---|---|---|---|---|
| | **1** | **2** | **3** | **4** | **5** | **6** | **R** | **T** |
| **1** | 46 | 33 | 34 | 34 | 31 | 31 | 35 | 35 |
| **2** | | 49 | 34 | 37 | 31 | 34 | 39 | 38 |
| **3** | | | 54 | 34 | 32 | 36 | 41 | 41 |
| **4** | | | | 51 | 30 | 35 | 38 | 39 |
| **5** | | | | | 44 | 32 | 37 | 37 |
| **6** | | | | | | 58 | 40 | 41 |
| **R** | | | | | | | 72 | 56 |
| **T** | | | | | | | | 70 |

The $(i, j)$th element of this table is the number of genes declared significant by both lab $i$ and lab $j$. R represents the results from a random effects meta-analysis, and T represents the 'true' number of genes that are differentially expressed. Each lab (including R) had the false discovery rate (FDR) controlled at 0.05.

useful in identifying genes that were statistically significantly differentially expressed, while taking into account the additional variation from the contributing labs. It did so without being overly influenced by any one lab that had potential to declare significance due to random variation. Rather than considering the union of all genes declared significant by any of the labs, and rather than simply taking the intersection of the lists of significant genes from each lab, the random effects meta-analysis combined information across all six labs in a well-structured manner and declared 72 genes significantly differentially expressed.

In order to gain an understanding of the success rate and power of the meta-analysis, a comparison of the results with the 'truth', or simulation parameters is performed. The numbers of correctly identified differentially expressed genes do not vary drastically between the individual labs (Table 1), but the meta-analysis tends to correctly identify a higher number of differentially expressed genes. In addition, the meta-analysis SLR estimates tend to be much closer to the true SLR values than do the estimates from individual labs (Figure 3). This demonstrates that the meta-analysis combines results from multiple labs to arrive at a more precise view of the 'true' degree of differential expression for each gene.

### Discussion

Using information from different laboratories interested in the same biological event, we have combined Affymetrix oligonucleotide arrays and the

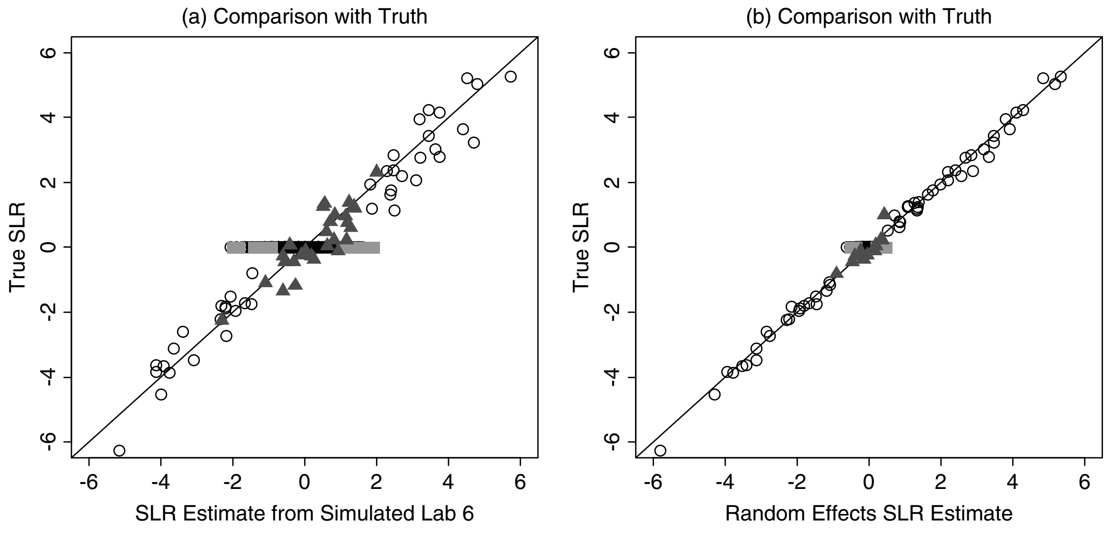

**Figure 3.** Comparison of SLR estimates with the 'true' SLR values. The SLR estimates from each lab (a) tend to approximate the true SLR values overall, but there is more deviation from the truth in each lab than there is in the random effects meta-analysis SLR estimates (b). Green squares represent type I errors, genes incorrectly claimed as differentially expressed. Red triangles represent type II errors, genes incorrectly claimed to be not significantly differentially expressed. There are fewer errors in the random effects meta-analysis results than there are in any given lab, and the random effects meta-analysis results tend to more closely approximate the true underlying degree of differential expression for each gene

Copyright © 2005 John Wiley & Sons, Ltd. 

results gained from Affymetrix software MAS 5.0 [2] for testing differential expression to demonstrate the utility and power of a meta-analytic approach. The signal log ratio (SLR), automatically reported by MAS 5.0 [2,23], is naturally suited to serve as an effect size estimate in a meta-analysis. The simulation example illustrated how the final SLR estimates from the meta-analysis models tend to be much closer to the 'true' SLR values than do the SLR estimates from any single lab. The application of meta-analytic approaches to microarray results provides a systematic method to combine results from different laboratories, with the purpose of gaining clearer insight into the true degree of differential expression for each gene. The random effects approach presented here can be extended to incorporate prior knowledge in a Bayesian framework, to account for known differences between experiments by use of covariates, and to adjust for possible dependencies between experiments. These extensions are the subject of ongoing and future work.

### Acknowledgements

We thank Dr Robert Meisel (Purdue University) and Dr Paul Mermelstein (University of Minnesota) for use of their RN_U34 Affymetrix data, which provided our simulation parameters.

### References

1. Affymetrix; **http://www.affymetrix.com**
2. *Affymetrix Microarray Suite User's Guide, Version 5.0*. 2001. Affymetrix: Santa Clara, CA.
3. Benjamini Y, Hochberg Y. 1995. Controlling the false discovery rate: a practical and powerful approach to multiple testing. *J R Stat Soc B* **57**(1): 289–300.
4. BioConductor: open source software for bioinformatics; **http://www.bioconductor.org**
5. Choi JK, Yu U, Kim S, Yoo OJ. 2003. Combining multiple microarray studies and modeling interstudy variation. *Bioinformatics* **19**(suppl 1): i84–i90. DOI: 10.1093/bioinformatics/btg1010
6. Cooper H, Hedges LV. 1994. *The Handbook of Research Synthesis*. Russell Sage Foundation: New York, NY.
7. Craig BA, Black MA, Doerge RW. 2003. Gene expression data: the technology and statistical analysis. *J Agric Biol Envir S* **8**(1): 1–28.
8. Cui XQ, Churchill GA. 2003. Statistical tests for differential expression in cDNA microarray experiments. *Genome Biol* **4**: 210.1–210.9.
9. Dudoit S, Yang YH, Speed TP, Callow MJ. 2002. Statistical methods for identifying differentially expressed genes in replicated cDNA microarray experiments. *Stat Sinica* **12**(1): 111–139.
10. DerSimonian R, Laird N. 1986. Meta-analysis in clinical trials. *Control Clin Trials* **7**: 177–188.
11. Gautier L, Cope L, Bolstad BM, Irizarry RA. 2004. affy-analysis of Affymetrix GeneChip data at the probe level. *Bioinformatics* **20**(3): 307–315. DOI: 10.1093/bioinformatics/btg405
12. Ghosh D, Barette TR, Rhodes D, Chinnaiyan AM. 2003. Statistical issues and methods for meta-analysis of microarray data: a case study in prostate cancer. *Funct Integr Genomics* **3**: 180–188. DOI: 10.1007/s10142-003-0087-5
13. Glass GV. 1976. Primary, secondary, and meta-analysis of research. *Educ Res* **10**: 3–8.
14. Glass GV. 1978. Integrating findings: the meta-analysis of research. *Rev Res Educ* **5**: 351–379.
15. Ihaka R, Gentleman R. 1996. R: a language for data analysis and graphics. *J Comp Graph Stat* **5**(3): 299–314.
16. Irizarry RA, Bolstad BM, Collin F, *et al*. 2003. Summaries of Affymetrix GeneChip probe level data. *Nucleic Acids Res* **31**: e15. DOI: 10.1093/nar/gng015
17. Moreau Y, Aerts S, De Moor B, De Strooper B, Dabrowski M. 2003. Comparison and meta-analysis of microarray data: from the bench to the computer desk. *Trends Genet* **19**(10): 570–577. DOI: 10.1016/j.tig/2003.08.006
18. Morris JS, Yin G, Baggerly K, Wu C, Zhang L. 2003. Identification of prognostic genes, combining information across different institutions and oligonucleotide arrays. Critical Assessment of Microarray Data Analysis (CAMDA) 2003 Conference Paper, available at **http://www.camda.duke.edu/camda03**
19. Parmigiani G, Garrett-Mayer ES, Anbazhagan R, Gabrielson E. 2004. A cross-study comparison of gene expression studies for the molecular classification of lung cancer. *Clin Cancer Res* **10**: 2922–2927.
20. Rhodes DR, Barrette TR, Rubin MA, Ghosh D, Chinnaiyan AM. 2002. Meta-analysis of microarrays: interstudy validation of gene expression profiles reveals pathway dysregulation in prostate cancer. *Cancer Res* **62**: 4427–4433.
21. Rhodes DR, Yu J, Shanker K, *et al*. 2004. Large-scale meta-analysis of cancer microarray data identifies common transcriptional profiles of neoplastic transformation and progression. *Proc Natl Acad Sci USA* **101**: 9309–9314. DOI: 10.1073/pnas.0401994101
22. Seber GAF. 1977. *Linear Regression Analysis*. Wiley: New York, NY.
23. *Statistical Algorithms Description Document*. 2002. Affymetrix: Santa Clara, CA.
24. Stevens JR, Doerge RW. 2005. Combining Affymetrix microarray results. *BMC Bioinformatics* **6**: 57.
25. The Comprehensive R Archive Network; **http://cran.r-project.org**
26. Wang J, Coombes KR, Highsmith WE, Keating MJ, Abruzzo LV. 2004. Differences in gene expression between B-cell chronic lymphocytic leukemia and normal B cells: a meta-analysis of three microarray studies. *Bioinformatics* **20**(17): 3166–3178. DOI: 10.1093/bioinformatics/bth381

Copyright © 2005 John Wiley & Sons, Ltd.
